# Overlapping Community Detection in Vehicular Social Networks Based on Graph Attention Autoencoder

**DOI:** 10.3390/s25082601

**Published:** 2025-04-20

**Authors:** Xiang Gu, Qiwei Huang, Jie Yang

**Affiliations:** 1 School of Artificial Intelligence and Computer Science, Nantong University, Nantong 226019, China; gu.x@ntu.edu.cn; 2 School of Information Science and Technology, Nantong University, Nantong 226019, China; 2330310006@stmail.ntu.edu.cn

**Keywords:** graph attention autoencoder, overlapping community detection, semi-supervised clustering, vehicular social networks

## Abstract

Community detection is particularly important in vehicular social networks because it helps identify closely connected groups of vehicles within the network. Community structures with overlapping relationships are identified through network topology and vehicle attribute information, thereby optimizing communication efficiency, supporting resource allocation, and enhancing privacy protection. However, most existing community detection methods focus on non-overlapping communities, usually only considering the topological structure of the network, and often ignoring the attribute information of nodes. To address these problems, this paper proposes a semi-supervised overlapping community detection method based on graph attention autoencoder (CDGAAE). The method consists of three key components: graph attention autoencoder module, modularity optimization enhancement module, and semi-supervised clustering module. First, the graph attention autoencoder module fuses topological information and node attribute information and encodes nodes using a graph attention mechanism. Second, the modularity optimization enhancement module effectively captures the structure of overlapping communities. Finally, the semi-supervised clustering module combines prior information to improve the accuracy of community detection. CDGAAE is comprehensively evaluated on multiple real and synthetic datasets, and experimental results show that CDGAAE outperforms other competing methods.

## 1. Introduction

With the development of intelligent transportation systems, vehicle social networks (VSNs) [1] have emerged as an important form of networking and are gradually becoming a research focus. This type of network utilizes communication between vehicles (V2V) and between vehicles and infrastructure (V2I) to improve road safety, optimize traffic flow, and enhance environmental monitoring capabilities. Community detection, which involves identifying densely connected groups of nodes in a network [2], is crucial for managing vehicles and maintaining security within vehicle social networks. These communities typically manifest as densely connected areas in the network, where the connections between nodes are strong and the connections to other communities are relatively sparse. Community detection has been widely applied in various fields, including social networks, biological networks, and citation networks. In social networks, it helps identify and analyze user groups, interest groups, and social circles [3]. In biological networks, community detection reveals functional modules in protein interaction networks [4]. In citation networks, it provides insights into the structure and distribution of academic fields [5]. In vehicle social networks, community detection can improve communication efficiency, enhance security and privacy protection, optimize resource allocation, and facilitate local management.

However, upon analyzing several reviews of community detection methods [6,7], we found that most approaches primarily focus on the topological structure of the network while neglecting the attribute information of the nodes. In vehicle social networks, community detection cannot rely solely on the topological structure; the social attributes of the vehicle (i.e., the driver’s social attributes) must also be considered to more accurately determine the community to which a vehicle node belongs. Furthermore, traditional community detection methods face challenges in extending to large-scale networks with high-dimensional attributes.

To address these challenges, researchers have proposed community detection methods that integrate network topology with node attribute information. For instance, CDBNE [8] introduces a framework that simultaneously learns representations based on network structure and attribute information, as well as clustering-oriented representations. In the context of large-scale networks that struggle to accommodate high-dimensional attributes, deep learning techniques are employed to process high-dimensional network data and learn low-dimensional representations of network structures [7]. For example, DNGR [9] utilizes a random surfing model to directly capture the structural information of the graph, generating a low-dimensional vector representation for each vertex while incorporating a stacked denoising autoencoder to extract complex features and model nonlinearity. Additionally, another study [10] proposes a novel modular non-negative matrix factorization (M-NMF) model that enables the learned node representation to retain both microstructure and community structure.

However, most existing community detection methods are designed for identifying non-overlapping communities, while overlapping communities are quite common in vehicular social networks. Vehicles may participate in different communities at various time periods or under different situations. Therefore, this paper proposes a semi-supervised overlapping community detection method, CDGAAE, based on a Graph Attention Autoencoder, which utilizes both topological structure and node attribute information to obtain a low-dimensional representation of the attribute network. To explore the potential relationships and interaction probabilities between nodes, we propose a modularity optimization enhancement. Additionally, we introduce a semi-supervised clustering module that guides the representation learning of nodes through prior information.

Our main contributions are summarized as follows:

- We design a Graph Attention Autoencoder that integrates topological structure and node attribute information through an encoder–decoder architecture to obtain a low-dimensional representation of the nodes;
- We propose an autoencoder-based overlapping community detection framework consisting of three modules: the Graph Attention Autoencoder, the modularity optimization enhancement module, and the semi-supervised clustering module. Training is conducted by jointly optimizing the graph structure and node attribute reconstruction loss, modularity optimization enhancement loss, and semi-supervised clustering loss;
- We conduct comprehensive tests on some real and synthetic datasets to evaluate the performance of CDGAAE relative to seven competitive baselines. Experimental results show that the proposed overlapping community detection model is effective.

The rest of this paper is organized as follows. Section 2 briefly summarizes related work. Section 3 defines the problem we aim to solve and describes the CDGAAE model in detail. In Section 4, we evaluate the experimental results of the proposed CDGAAE across several datasets. Finally, Section 5 concludes the paper and discusses future research directions.

## 2. Related Work

There are three main methods for fusing topological information and attribute information for community detection. The first is early fusion methods, which integrate structural and attribute information before the community detection process, ensuring that the resulting data are suitable for classic community detection algorithms. Representative methods include NMLPA [11], SToC [12], SCMAG [13], CDE [14], and AHMotif [15]. The second method is simultaneous fusion, which utilizes both structural and attribute information during the community detection process. Representative methods include kNAS [16], MOEA-SA [17], ASCD [18], COMODO [19], NEMBP [20], and SLA [21]. The third method is late fusion, which aims to combine structural and attribute information after the community detection process. Representative methods include WCMFA [22], Multiplex [23], and Selection [24].

The autoencoder [25] is an unsupervised learning algorithm designed to learn effective data representations through encoding and decoding processes. It plays a vital role in community detection. For instance, AAGR [26] and DIR [27] employ stacked autoencoders to adaptively fuse topology and attribute information, introducing a novel adaptive parameter to achieve robust integration of structural and content information. DFuzzy [28] utilizes sparse autoencoders as building blocks and performs fine-tuning to learn latent representations by mining local information and training the autoencoder using PageRank. MAGE [29] proposes a marginalized graph convolutional network that corrupts network node content, allowing node content to interact with network features, and marginalizes the corrupted features within the graph autoencoder environment to learn graph feature representations. During the encoding stage, NetVAE [30] employs the same encoder for compressing both network structure and node attributes, performing joint training to facilitate transfer learning and information integration. In the decoding stage, dual decoders are introduced to reconstruct network topology and node attributes, respectively.

Guo et al. proposed an attention-walking autoencoder (AWBA [31]). Not only did they add an attention layer to the encoder to learn the influence of different neighbors of a node in the encoding process, but they also developed a new random walk strategy that combines the node neighbor information provided by the attention coefficient and the community membership of the node to embed low-order and high-order node relationships into the representation vector, thereby achieving high-quality communities. Prokop et al. proposed a hierarchical overlapping community detection method [32] with HAC and maximum clique as clustering primitives. A new dissimilarity measure for graph hierarchical agglomerative clustering (GHAC) was introduced to detect overlapping communities in networks. Li et al. proposed a dual graph neural network (DGOCD [33]) for overlapping community detection under the framework of the extended Bernoulli–Poisson model. By constructing two graphs, the information of different orders between nodes is modeled, and then the concept of topological potential matrix is introduced to aggregate the embedding representation of the two channel graphs. The network is reconstructed based on the previous information, and the reconstructed network is fed into GCN to obtain the final community division. Zhou et al. proposed an overlapping community detection model LQ-GCN [34] based on the local community perspective. The Bernoulli–Poisson model was used to construct the community membership matrix and an end-to-end detection framework was constructed. The model uses local modularity as the objective function and incorporates local community information to improve the quality and accuracy of clustering results.

Although existing methods can fuse topological information and attribute information to some extent, most of these methods require manual adjustment of hyperparameters to balance the two types of information. For instance, CODICIL [35] necessitates manual setting of weight parameters to construct a similarity fusion matrix for subsequent community detection. Additionally, most existing methods address overlapping community detection and the integration of prior information separately. For example, the semi-supervised GCN community detection model MRFasGCN [36] incorporates prior information to characterize hidden communities by extending the network-specific Markov random field (eMRF) into a new convolutional layer. Similarly, NOCD [37] is designed for overlapping community detection; this model combines the Bernoulli–Poisson (BP) probability model with two layers of GCN to learn the community belonging vector by minimizing the negative log-likelihood of BP.

According to the literature survey, there is currently a lack of overlapping community detection methods that effectively utilize prior information while fusing topological and attribute information. This paper proposes the CDGAAE method, which not only automatically and seamlessly integrates topological and attribute information but also leverages prior information to detect overlapping communities. Compared to existing overlapping community detection methods, CDGAAE is expected to be more effective and generalizable.

## 3. Proposed Method

In this section, the CDGAAE approach is described in full in this part after we first introduce the fundamental terms and symbols utilized in this work.

### 3.1. Preliminary Knowledge and Problem Statement

**Definition 1** (Vehicle Social Network). *Given a vehicle social network G=(V,E,X,A), where $V=v_{1},v_{2},\ldots ,v_{n}$ represents a set of nodes, and n=|V| is the number of nodes. $E=\left(v_{i},v_{j}\right)|v_{i},v_{j}\in V,A_{ij}=1$ represents the set of edges. For any two nodes $v_{i},v_{j}\in V$, if there is an edge $e_{ij}\in E$, it means that there is a connection between nodes $v_{i}$ and $v_{j}$. $X=x_{1},x_{2},\ldots ,x_{n}$ represents the set of node attributes, where $x_{i}\in R^{d}$ is the attribute vector of node $v_{i}$ and d is the dimension of the attributes. $A\in R^{n\times n}$ represents the adjacency matrix of the network, where $A_{ij}$ represents the connection between nodes $v_{i}$ and $v_{j}$:*

(1) $$A_{ij}=\left\{\begin{array}{c} 1,(v_{i},v_{j})\in E\\ 0,(v_{i},v_{j})\notin E \end{array}\right.$$

**Definition 2** (Community). *A community is a set of nodes in a network. Given a network G=(V,E), a community $C_{k}$ can be defined as a subset $C_{k}\subseteq V$. The connections between nodes within this set are relatively dense, while the connections between nodes in different sets are relatively sparse. In other words, the density of internal connections is high, and the density of external connections is low.*

**Definition 3** (Prior Information). *Since certain nodes are labeled with their true community labels, we treat known community label information as prior information. The precision and efficacy of community discovery can be greatly increased by encoding this data into a prior node community label matrix.*

**Problem statement:** Given a vehicle social network *G*, which contains topological information *A*, attribute information *X*, and prior information *Y*, the input is the topological structure of the vehicle network (adjacency matrix *A*) and the node attribute matrix *X*. Community detection aims to identify *k* communities, where overlapping communities are allowed. Nodes in the same community are closely connected and have similar characteristics.

### 3.2. Overview of the Overall Model

**Figure 1:**
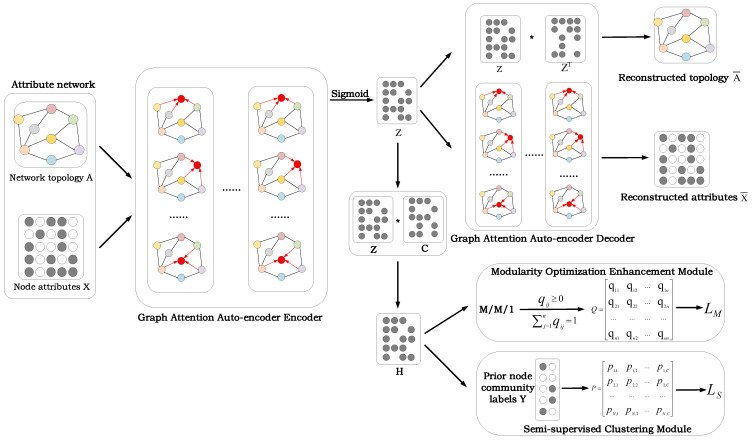
The framework of the CDGAAE model.

In this section, we propose the CDGAAE model for overlapping community detection in vehicular social networks. The overall framework is illustrated in Figure 1.

As illustrated in the figure, the framework comprises three components: the graph attention autoencoder, modularity optimization enhancement, and a semi-supervised clustering module. (1) Graph Attention Autoencoder: This component employs a graph attention mechanism to encode both the topological structure and attribute information, producing a low-dimensional representation of the attribute network by minimizing the reconstruction loss of these features. The encoder learns effective representations of the input data, while the decoder reconstructs the original input features. (2) Modularity Optimization Enhancement Module: A Markov queuing model is integrated with traditional modularity maximization to more accurately simulate potential relationships and interaction probabilities between nodes. (3) Semi-Supervised Clustering Module: Using prior information constraints, this module effectively captures complex relationships between nodes, enhancing the accuracy of community detection. Finally, the model is trained by a unified loss function *L* consisting of the graph structure and node attribute reconstruction loss $L_{R}$, the modularity optimization enhancement loss $L_{M}$, and the semi-supervised clustering loss $L_{S}$. Next, we introduce CDGAAE in detail.

The proposed CDGAAE aims to achieve efficient overlapping community discovery in in-vehicle social networks by combining graph attention autoencoders, module optimization enhancement, and semi-supervised learning. The model takes an attribute network as input, where the network topology *A* represents the connection relationship between nodes and the node attribute matrix *X* contains the multi-dimensional feature information of each node. The input data first pass through the encoder module of the graph attention autoencoder, which captures the characteristics and global topological information of neighborhood nodes through the attention mechanism and generates high-quality node embedding representation *Z*. Subsequently, the decoder module uses *Z* to reconstruct the network topology $\hat{A}$ and node attributes $\hat{X}$ to minimize the reconstruction loss $L_{R}$, ensuring that the embedding can preserve the structure and attribute information of the input data. In order to improve the global consistency of community division and ensure that the embedding can better reflect the community structure of the network, the model introduces a module optimization enhancement module. This module improves the consistency of the global community structure by optimizing the modularity value *Q* and introduces a Markov queuing model to effectively capture the potential connections and interaction probabilities between nodes. In addition, the model also combines a semi-supervised clustering module, which uses the supervised information provided by a small amount of prior labels *Y* to calculate the semi-supervised loss $L_{S}$, thereby guiding the community division result *H* to more accurately match the actual community structure.

The main symbols and their related explanations are shown in Table 1.

### 3.3. Graph Attention Autoencoder

The graph attention autoencoder enhances representation learning in the graph by introducing an attention mechanism, which can handle topological and attribute information more flexibly. The graph attention mechanism can capture the high-dimensional nonlinear relationship between vehicle nodes and represent the potential distribution of vehicle nodes in community partitioning through an embedding matrix. Through its autoencoder structure, the model can not only learn the latent representation of the graph, but also optimize it using reconstruction loss to better capture key structural features. The module consists of two components: an encoder and a decoder. The encoder’s graph attention layer summarizes and updates the features of each node and outputs the hidden layer representation of each node. The decoder’s task is to construct the head information matrix and node attribute information from the latent space representation. The reconstruction loss $L_{R}$ ensures the fidelity of the embedding to the network topology and node attributes.

#### 3.3.1. Encoder

Although single-head attention can capture relationships between nodes, it is limited by a single weight vector and may not fully capture the complex patterns and features within the graph. Multi-head attention addresses this limitation by using multiple attention heads, enabling the model to aggregate features and model relationships from multiple perspectives.

In the multi-head attention mechanism, we introduce *l* independent attention heads, each with its own linear transformation matrix $W^{\left(l\right)}$ to project the original node features into a new feature space. This transformation is applied to each node feature vector $h_{i}$ individually, and the formula is as follows:

(2) $$h_{i}^{\left(l\right)}=W^{\left(l\right)}h_{i}$$

where $h_{i}^{\left(l\right)}$ is the new feature representation of node $v_{i}$ in the *l*th head. Then, an attention score is computed for each pair of nodes $v_{i}$ and $v_{j}$, indicating the importance of node $v_{j}$ to node $v_{i}$. This score is calculated based on the sum of their linearly transformed feature representations. The specific formula is

(3) $$e_{ij}^{\left(l\right)}=\mathrm{LeakyReLU}\left(a^{(l)⊤}\left[W^{\left(l\right)}h_{i}∥W^{\left(l\right)}h_{j}\right]\right)$$

where $a^{\left(l\right)}$ is the attention weight vector of the *l*th head, with a length of $2F^{\prime }$, used to map the concatenated vectors to a scalar. The symbol || denotes the concatenation operation, and $F^{\prime }$ is the dimension of the new feature space for each head. LeakyReLU is the activation function, typically chosen with a small negative slope, such as α=0.2. It is defined as

(4) $$\mathrm{LeakyReLU}(x)=\left\{\begin{array}{cc} x, & \mathrm{if}\;x\geq 0\\ \alpha x, & \mathrm{if}\;x<0 \end{array}\right.$$

We use the softmax function to normalize the raw attention scores of all neighboring nodes to obtain the final attention coefficient $\alpha_{ij}^{\left(l\right)}$. This process ensures that the sum of the attention coefficients for all neighboring nodes equals 1:

(5) $$\alpha_{ij}^{\left(l\right)}=\frac{\exp(e_{ij}^{\left(l\right)})}{\sum_{l\in N(i)}\exp\left(e_{il}^{\left(l\right)}\right)}$$

For each attention head *l*, we weigh and sum the neighboring node features using the attention coefficients $\alpha_{ij}^{\left(l\right)}$ to obtain the feature output $h_{i}^{\left(l\right)}$ for each head *l*:

(6) $$h_{i}^{\left(l\right)}=\sum_{j\in N(i)}\alpha_{ij}^{\left(l\right)}W^{\left(l\right)}h_{j}$$

The output features of all heads are averaged to obtain the final node representation:

(7) $$h_{i}^{\prime }=\frac{1}{L}\sum^{L}h_{i}^{\left(l\right)}$$

#### 3.3.2. Decoder

The decoder is responsible for reconstructing the graph structure from the latent representations generated by the encoder, typically by reconstructing the adjacency matrix. The main task is to reconstruct the graph’s adjacency matrix $\hat{A}$ from the latent node representations *Z*, where $\hat{A}$ is the predicted adjacency matrix. When using the multi-head attention mechanism, the latent representation $z_{i}$ is typically aggregated from the output features $z_{i}^{\left(l\right)}$ of multiple attention heads. If we average the output features of multiple heads, then the latent representation of each node is

(8) $$z_{i}=\frac{1}{L}\sum_{l=1}^{L}z_{i}^{\left(l\right)}$$

At this point, the latent representation of the node $z_{i}$ is still an $F^{\prime }$-dimensional vector. The formula for the decoder remains the same,

(9) $${\hat{A}}_{ij}=\sigma \left(z_{i}^{⊤}z_{j}\right)$$

where ${\hat{A}}_{ij}$ represents the existence of an edge between node *i* and node *j* predicted by the decoder, and $z_{i}$ and $z_{j}$ are the latent representations of node *i* and node *j*, respectively. The function σ is the activation function, typically chosen as the Sigmoid function:

(10) $$\sigma \left(x\right)=\frac{1}{1+\exp(-x)}$$

The final result of the decoder is used as the reconstructed node attribute value and the loss function for reconstructing the node attribute value is defined as Equation (11).

(11) $$L_{attr}=-\frac{1}{n}\sum_{i=1}^{n}\left[X_{i}\log{\hat{X}}_{i}+\left(1-X_{i}\right)\log\left(1-{\hat{X}}_{i}\right)\right]$$

where $X_{i}$ is the original binary attribute of the *i*th node and ${\hat{X}}_{i}$ is the predicted probability that the attribute of the *i*th node is 1. The loss function computes the binary cross-entropy for the binary attribute reconstruction of each node and optimizes the model’s reconstruction of node attributes by minimizing this loss.

The loss function for neighbor matrix prediction is defined as Equation (12).

(12) $$L_{top}=-\sum_{i,j}\left(A_{ij}\log{\hat{A}}_{ij}+\left(1-A_{ij}\right)\log\left(1-{\hat{A}}_{ij}\right)\right)$$

where $A_{ij}$ represents an element in the original adjacency matrix, indicating whether there is an edge between node *i* and node *j*. If $A_{ij}=1$, it means there is an edge between node *i* and node *j*, and if $A_{ij}=0$, it means there is no edge. ${\hat{A}}_{ij}$ is the element in the predicted adjacency matrix, representing the probability that the model predicts an edge between node *i* and node *j*.

The final reconfiguration loss function is formed by the combination of graph structure reconfiguration loss and node attribute reconfiguration loss.

(13) $$L_{R}=\alpha L_{attr}+\beta L_{\mathrm{top}}$$

where α and β are hyperparameters used to balance the effects of node attribute reconstruction loss and neighbor matrix prediction loss.

### 3.4. Modularity Optimization and Enhancement Module

When combining topological and attribute information to divide overlapping communities, maximizing modularity optimizes the quality of the division [38], resulting in a community structure with higher modularity. This ensures that connections between nodes within the same community are dense, while connections between different communities are sparse. Modularity, a measure of the strength of network community structures [39], can be defined as

(14) $$Q=\frac{1}{4m}\sum_{i,j}\left(A_{ij}-\frac{d_{i}d_{j}}{2m}\right)\left(H_{i}H_{j}^{⊤}\right)$$

where $A_{ij}$ is the adjacency matrix, which represents the connection relationship between nodes *i* and *j* (1 means there is an edge, 0 means no edge). 2m is the total number of edges in the network. $d_{i}$ and $d_{j}$ are the degrees of node *i* and node *j*, respectively, $H_{i}H_{j}^{⊤}$ is the community similarity measure of nodes *i* and *j*, which calculates their consistency in community division.

We incorporated a Markov queuing system [40] into the modularity maximization module, transforming it into a modularity optimization enhancement module. In the traditional modularity maximization method, nodes are rigidly assigned to a single community. By introducing the Markov queuing system, nodes can naturally flow across multiple communities, allowing for a more accurate representation of overlapping community structures. Additionally, interactions between nodes are often uncertain. The Markov queuing system effectively captures these uncertainties using a probabilistic model, which more accurately reflects the likelihood of nodes belonging to different communities.

In a Markov queuing system, the state transitions between nodes can be represented by the state transition matrix *P*. This matrix describes the transition probability between node $v_{i}$ and node $v_{j}$ as $P_{ij}$. Based on this, we can incorporate the state transition probabilities into the optimization formulation of the modularity, thereby enhancing the capture of dynamic interactions between nodes. We define the enhanced modularity optimization objective as

(15) $$Q_{M}=\frac{1}{4m}\sum_{i,j}\left(A_{ij}P_{ij}-\frac{d_{i}d_{j}P_{ij}}{2m}\right)\left(H_{i}H_{j}^{⊤}\right)$$

where $P_{ij}$ denotes the probability of node *i* transferring to node *j* and $A_{ij}P_{ij}$ represents the strength of dynamic interactions between nodes, considering not only the presence of direct edges between nodes but also the strength of these dynamic interactions.

The modularity optimization objective can be viewed as a regularization term for GAE embedding, guiding embedding learning to be more consistent with the goal of community segmentation. We define the modularity optimization loss as

(16) $$L_{M}=-Q_{M}=-\frac{1}{4m}\sum_{i,j}\left(A_{ij}P_{ij}-\frac{d_{i}d_{j}P_{ij}}{2m}\right)\left(H_{i}H_{j}^{⊤}\right)$$

The objective function $L_{M}$ clarifies the community division and enhances the ability to capture the global structure of the network. In order to combine the objectives enhanced by module optimization, we need to propagate the gradient of $L_{M}$ to the embedding. *H* can be updated by taking the gradient of the modularity:

(17) $$\frac{\partial L_{M}}{\partial H_{i}}=-\frac{1}{4m}\sum_{i,j}\left(A_{ij}P_{ij}-\frac{d_{i}d_{j}P_{ij}}{2m}\right)H_{j}^{⊤}$$

This means that for each node *i*, its community representation matrix $H_{i}$ is adjusted so that it is closer to the community representation $H_{j}$ of the node *j* with which it has a stronger connection (i.e., $A_{ij}P_{ij}>\frac{d_{i}d_{j}P_{ij}}{2m}$).

### 3.5. Semi-Supervised Clustering Module

The semi-supervised clustering module does this by introducing a portion of known node labels (e.g., the community affiliation of certain nodes is known) as a prior information. This labeling information can significantly improve the clustering performance of the model as it provides direct guidance on the correct community delineation. With a semi-supervised strategy, it is possible to maintain the consistency of the known labels and simultaneously mine the potential community affiliations of unlabeled nodes, making the overall community division more rational. Therefore, we can apply the widely used cross-entropy loss as a semi-supervised loss $L_{S}$:

(18) $$L_{S}=-\sum_{i\in Y_{j}=1}^{k}Y_{ij}\ln H_{ij}$$

where Y is the set of labeled data, and $Y_{ij}$ denotes that node *i* belongs to community *j* based on the ground truth labels. The semi-supervised loss $L_{S}$ integrates the prior community labels into the optimization objective, improving the accuracy of overlapping community detection. As the number of labeled nodes in Y increases, the supervision signal of the model is enhanced, and the optimization objective converges to the global optimal solution more easily. In theory, as the proportion of prior information increases, the performance improvement of the model gradually decreases. This is because when the proportion of prior information is low, the model relies more on labeled information, and adding labeled nodes can significantly improve performance. When the proportion is high, the community division of the model is stable enough, and the incremental information provided by the newly added labeled nodes is limited.

In summary, using a graph attention autoencoder alone is not sufficient to obtain a more accurate representation matrix of community members *H*. By integrating the graph attention autoencoder, modularity optimization enhancement, and a semi-supervised clustering module into a unified framework, we can achieve more accurate community detection results. For this purpose, the final objective function of CDGAAE is defined in Equation (19).

(19) $$L=L_{R}+\gamma L_{S}-\lambda L_{M}$$

where γ and λ are hyperparameters that represent different loss weights to adjust the contributions of the corresponding modules. $L_{R}$, $L_{M}$, and $L_{S}$ represent the losses of the graph attention autoencoder, modularity optimization enhancement, and self-training clustering modules, respectively.

### 3.6. Overlapping Community Detection Algorithm and Complexity Analysis

Upon completion of training, CDGAAE generates a stabilized matrix of community member representations *H*. To detect overlapping communities, one can refer to Yang and Leskovec’s method [41] to determine the community affiliation of a node by setting a threshold value φ. Specifically, when $H_{ip}$ is greater than or equal to φ, node $v_{i}$ is considered to belong to community $C_{p}$. This method is simple and effective. To determine φ rationally, we compute it as

(20) $$\varphi =\sqrt{-\log\left(1-\frac{2m}{n(n-1)}\right)}$$

where *n* represents the number of nodes, *m* represents the number of edges.

The threshold segmentation strategy is effective in the actual overlapping community detection. Based on the above idea, we design a complete CDGAAE overlapping community detection algorithm, which is given in Algorithm 1.
**Algorithm 1** CDGAAE Overlapping Community Detection**Input:** Vehicle social network G=(V,E,X,A), Prior information *Y*, Number of communities *k*, Maximum iterations MaxIter;**Output:** Communities set $C=\{C_{1},C_{2},\ldots ,C_{k}\}$;1Initialize attention weights $W^{\left(k\right)}$ in GAAE using Xavier initialization2**for** t=1 to MaxIter **do**3      Generate $\alpha_{ij}$ and *H* with GAAE encoder4      Feed representations *H* to GAAE decoder to reconstruct adjacency matrix $\hat{A}$5      Calculate $L_{R}$, $L_{M}$, and $L_{S}$, respectively6      Calculate unified loss function *L* via Equation (18)7      Backpropagate and update attention weights $W^{\left(k\right)}$ in GAAE8**end for**9**for** each node $v_{i}\in V$ **do**10     **for** each $H_{ip}\in H_{i}$ **do**11            **if** $H_{ip}\geq -\log\left(1-\frac{2m}{n(n-1)}\right)$ **then**12                   $C_{p}=C_{p}\cup \left\{v_{i}\right\}$13            **end if**14      **end for**15**end for**16**return** the community set *C*

As shown in Algorithm 1, calculating the attention coefficient $\alpha_{ij}$ typically involves a weighted dot product operation. The number of nodes is *n* and the number of edges is *m*. In the worst case, all nodes are connected to each other, requiring the calculation of attention coefficients for $n^{2}$ pairs. Therefore, the time complexity for this part is $O(n^{2})$. The decoding step is performed through matrix multiplication, resulting in a time complexity of $O(n^{2}\times d)$, where *d* is the dimension of the node features. The graph reconstruction loss $L_{R}$ has a time complexity of $O(n^{2})$, the modularity optimization enhancement loss $L_{M}$ has a time complexity of O(n), and the semi-supervised clustering loss $L_{S}$ also has a time complexity of O(n). The computation of the overall loss function is the weighted sum of these three losses, hence the time complexity is O(1). For node assignment, in the worst case, nodes can belong to all communities, resulting in a time complexity of O(n×k), where *k* is the number of communities. Overall, the time complexity of CDGAAE is $O(\mathrm{MaxIter}\times \left(n^{2}\times d\right)+n\times k)$.

## 4. Experiments

To verify the effectiveness of CDGAAE, we first conduct comparative analysis with four baseline methods and seven baseline methods on various synthetic and real datasets, demonstrating the superior performance of CDGAAE. We then conduct ablation studies on the graph attention autoencoder module, modularity optimization enhancement module, and semi-supervised clustering module, and finally discuss the process of parameter and dimension selection.

### 4.1. Dataset

Since vehicles in a vehicle social network are controlled by drivers, studying the social attributes of vehicle nodes effectively means studying the social behavior of the drivers. Therefore, community detection in a vehicle social network aims to identify potential driver communities. For our experiments, we use real-world social network datasets from Facebook, including Facebook414, Facebook1684, and Facebook1912. A summary of the experimental datasets is provided in Table 2.

The Facebook dataset contains only the social attributes of individuals and lacks any associated vehicle attributes. To address this, we randomly add vehicle attributes to the three Facebook datasets mentioned above to create new synthetic datasets.

### 4.2. Evaluation Metrics

We introduce three widely used evaluation metrics for assessing the detection accuracy of overlapping communities: Overlapping Normalized Mutual Information (ONMI) [42], Average F1 Score (F1 for short) [41], and the Jaccard Index [43].

Overlapping Normalized Mutual Information (ONMI) is a metric used to evaluate the results of overlapping community detection. As an extension of the standard Normalized Mutual Information (NMI), it accounts for cases where a node belongs to multiple communities simultaneously. The value range of ONMI is [0, 1]. The closer the value is to 1, the closer the community division result is to the real community division. The calculation formula is as follows:

(21) $$ONMI=1-\frac{1}{2}\left(\frac{1}{|C|}\sum^{\left|C\right|}\frac{H(C_{i}|C^{\prime })}{H(C_{i})}+\frac{1}{|C^{\prime }|}\sum^{|C^{\prime }|}\frac{H(C_{i}^{\prime }|C)}{H(C_{i}^{\prime })}\right)$$

where C represents the actual community partition set, $C^{\prime }$ represents the detected community partition set, and $H(C_{i})$ is the entropy of community $C_{i}$. The calculation formula is as follows:

(22) $$H\left(C_{i}\right)=-\sum_{j=1}^{\left|C\right|}P\left(C_{i}\right)\log P\left(C_{i}\right)$$

where $P(C_{i})$ is the probability distribution of a node belonging to community $C_{i}$. $H(C_{i})$ measures the degree of concentration of node distribution within a community. A higher entropy value indicates that the distribution of community divisions is more dispersed and the uncertainty is higher; a lower entropy value indicates that the community divisions are more concentrated and the certainty is higher. And $H(C_{i}|C^{\prime })$ represents the conditional entropy of the community $C_{i}$ under the condition of known detection community set $C^{\prime }$:

(23) $$H\left(C_{i}|C^{\prime }\right)=-\sum_{j=1}^{|C^{\prime }|}P\left(C_{i}|C_{j}^{\prime }\right)\log P\left(C_{i}|C_{j}^{\prime }\right)$$

where $P(C_{i}|C_{j}^{\prime })$ is the probability that a node belongs to community $C_{i}$, conditional on the detected community $C_{j}^{\prime }$. The conditional entropy $H(C_{i}|C^{\prime })$ is used to measure the dependency between the community $C_{i}$ and the detection result $C^{\prime }$. A lower conditional entropy means that $C_{i}$ can be better explained by the detection community $C^{\prime }$.

The F1 score is the harmonic mean of precision and recall, and its formula is as follows:

(24) $$F1=\frac{1}{2}\left(\frac{1}{|C^{\prime }|}\sum_{i}F1\left(C_{i}^{\prime },C_{g(i)}\right)+\frac{1}{|C|}\sum_{i}F1\left(C_{i},C_{g(i)}^{\prime }\right)\right)$$

where $C_{g(i)}$ represents the community in the test community set $C^{\prime }$ that best matches with $C_{i}$ in the true community set, and $C_{i}$ represents the community in the true community set that best matches with $C_{g(i)}^{\prime }$ in the test community set. The first half of the formula calculates the F1 score between each community in the true community set $C_{i}$ and the best matching community in the test community set $C_{g(i)}^{\prime }$, while the second half calculates the F1 score between each community in the test community set and the best matching community in the true community set. This calculation method considers both the matching from the true community to the test community and from the test community to the true community.

The Jaccard Index is a metric that measures the similarity between two sets. It is defined as the size of the intersection of the two sets divided by the size of their union. The calculation formula for the Jaccard Index is as follows:

(25) $$J=\frac{1}{2}\left(\frac{1}{|C^{\prime }|}\sum_{i}J\left(C_{i},C_{g(i)}\right)+\frac{1}{|C|}\sum_{i}J\left(C_{i},C_{g^{\left(i\right)}}^{\prime }\right)\right)$$

Similar to the calculation of the F1 score, the Jaccard Index also considers the similarity between each true community and its most closely matching detected community, as well as between each detected community and its most closely matching true community.

In order to prove the superiority of the proposed CDGAAE method, we use the above three evaluation indexes to run each method for 10 times and take the average result.

### 4.3. Baselines

We compare CDGAAE with seven competing methods, including unsupervised and semi-supervised methods, and four competing methods, depending on whether or not to integrate prior information.

**CESNA** [44]. CESNA developed an algorithm based on edge structure and node attributes, simulating the interaction between network structure and node attributes to more accurately detect overlapping communities in networks with node attributes.

**SCI** [45]. The SCI method combines topology-based community membership and node-attribute-based community attributes in a non-negative matrix factorization framework while introducing a sparsity penalty to select the most relevant attributes for each community and proposing an efficient update rule to evaluate parameters with convergence guarantees.

**NOCD** [37]. The core idea of NOCD is to combine GNNS with the Bernoulli–Poisson probability model. At the same time, this method can seamlessly merge the node features into the model, which is more convenient for the detection of overlapping communities.

**Bespoke** [46]. Bespoke proposed a semi-supervised community detection algorithm that models the structural composition of known communities by computing fingerprints based on the structural features of nodes and edges in known communities. It then extracts size and structural composition patterns in known communities and uses them to search for similar communities on the full graph.

**SSGCAE** [47]. SSGCAE is an overlapping community detection method based on a graph neural network. The method consists of three modules: graph convolutional autoencoder (GCAE) is used to fuse link information and attribute information, semi-supervised is used to integrate previous information, and modularity maximization is used to detect overlapping communities.

**CPGC** [48]. CPGC is a new community detection method based on community perspective and a graph convolutional network, which solves these limitations in attribute networks without previous label information. By directly generating the membership matrix using the B-P model, the fusion of representation learning and clustering is achieved. A modularity-based objective function is also introduced to improve the quality of the detected community.

**OCDGAN** [49]. OCDGAN is a new attention-based overlapping community detection method that incorporates the attention mechanism into the well-known Neural Overlapping Community Detection (NOCD) method to discover overlapping communities in graphs. This method studies whether the attention mechanism can discover the difference in input importance, so that the model can pay more attention to important nodes in the neighborhood.

### 4.4. Analysis of Experimental Results

In this section, we first perform comparative experiments on both real and synthetic datasets, running CDGAAE 10 times on each dataset to obtain the average values of ONMI, F1, and the Jaccard Index, demonstrating the superiority of CDGAAE. Next, we conduct an ablation study to confirm that the map attention automatic encoder module, modular optimization enhancement module, and semi-supervised clustering module play a key role in revealing the precise overlapping community structure. Finally, the parameters of CDGAAE are analyzed.

#### 4.4.1. Real Dataset

**Figure 2:**
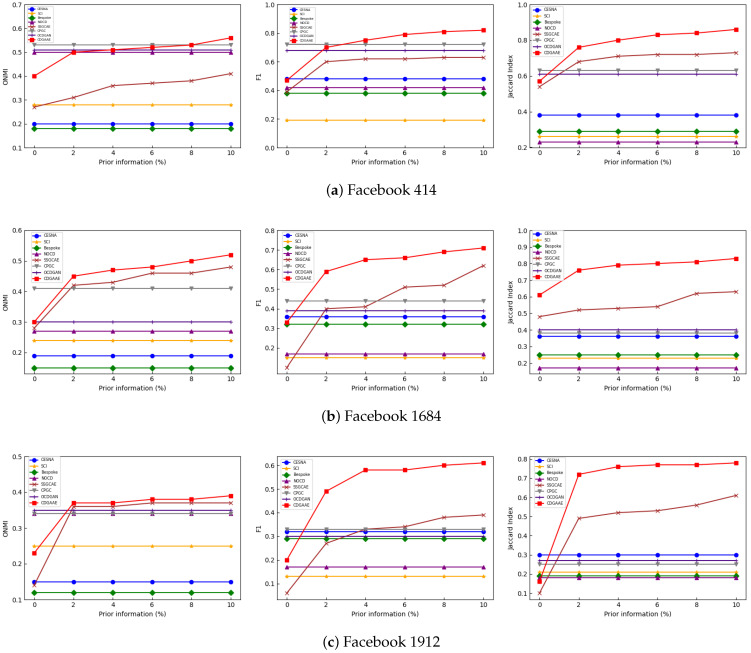
Performance comparison of ONMI, F1 and Jaccard indexes with the proportion of prior information on real datasets by different methods.

First, we test the performance of each method on three real datasets. The proportion of prior information ranges from 0% to 10%, and each increment is 2%. The experimental results are shown in Figure 2 below. As shown in the figure, as the proportion of prior information increases, the performance of CDGAAE on each dataset gradually improves. Based on these results, we can draw the following conclusions.

(1) CDGAAE outperforms unsupervised methods such as CPGC and OCDGAN, demonstrating the effectiveness of incorporating prior information into our method. As the proportion of prior information increases, the performance of CDGAAE gradually outperforms these unsupervised methods.
(2) CDGAAE outperforms the semi-supervised SSGCAE method, highlighting the importance of extracting community structure and reconstructing relevant attribute features. CDGAAE employs a graph attention mechanism to dynamically assign different weights to each node and uses an optimized modularity metric to generate node representations, which aids in producing topologically cohesive community results.
(3) We observe that the performance improvement of CDGAAE varies across datasets and evaluation metrics. On simpler networks such as Facebook 414, traditional methods already perform well with limited room for improvement. In contrast, for more complex datasets such as Facebook 1684 and Facebook 1912, CDGAAE achieves smaller improvements on ONMI but significant improvements in F1 score and Jaccard index, reflecting the improvement in node-level accuracy of overlapping community detection. This suggests that CDGAAE performs particularly well on networks with complex and ambiguous structures, where capturing fine-grained community associations is critical.

#### 4.4.2. Synthetic Dataset

**Figure 3:**
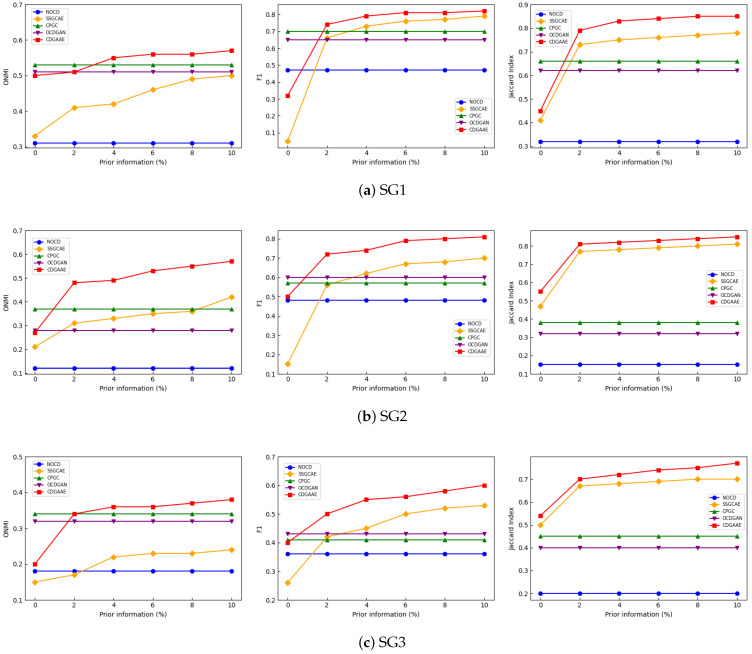
Performance comparison of ONMI, F1, and Jaccard indexes with the proportion of prior information on synthetic datasets by different methods.

According to previous experiments, we test the performance of each method on a synthetic dataset with added vehicle attributes, varying the proportion of prior information from 0% to 10% with an increment of 2%. The baselines used are the unsupervised methods NOCD, CPGC, OCDGAN, and the semi-supervised method SSGCAE. The results are shown in Figure 3. Similar to the results on the real dataset, the performance of CDGAAE improves with the increase in the proportion of prior information, outperforming NOCD, CPGC, OCDGAN, and SSGCAE.

#### 4.4.3. Ablation Experiment

As introduced in Section 3.3, Section 3.4 and Section 3.5, the graph attention autoencoder encodes nodes by introducing an attention mechanism to obtain high-quality node embedding representations. The main goal of the modular optimization enhancement module is to improve the quality of the community structure and ensure that the detected communities are more closely aligned with the actual community divisions. The core function of the semi-supervised clustering module is to use a small amount of label information to guide community divisions, thereby improving detection accuracy. In order to further verify the effectiveness of modular optimization enhancement and semi-supervised clustering in CDGAAE, we conduct ablation experiments and define the following three variants:

(1) CDGAAE_gcae: CDGAAE without the attention mechanism, only witha traditional graph convolutional autoencoder module. In this variant, node representation is obtained by fusing topological information and attribute information only through a traditional graph convolutional autoencoder; community structure is enhanced by the modularity optimization enhancement module and community detection is performed by the semi-supervised clustering module.
(2) CDGAAE_mod: CDGAAE without the modularity optimization enhancement module. In this variant, node representations are processed solely through the graph attention autoencoder and the semi-supervised clustering module for community detection.
(3) CDGAAE_unsup: CDGAAE without the semi-supervised clustering module. In this variant, node representations are processed using only the graph attention autoencoder and the modularity optimization enhancement module, adopting an unsupervised approach to community detection.

**Figure 4:**
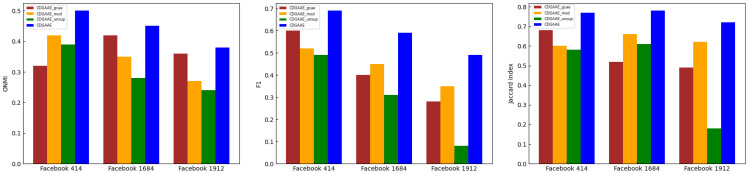
Performance comparison between CDGAAE, CDGAAE_gcae, CDGAAE_mod, and CDGAAE_unsup on real dataset.

We evaluate CDGAAE on three real datasets, each with 2% prior information. The results, shown in the Figure 4, clearly highlight the contributions of the two modules to the overall performance. For example, compared with CDGAAE_gcae on Facebook 414, CDGAAE improves ONMI, F1, and Jaccard index by 18%, 10%, and 8%, respectively, proving that the introduction of the attention mechanism can better learn node representations and thus improve the accuracy of community detection. Compared with CDGAAE_unsup on Facebook 1684, CDGAAE improves ONMI, F1, and the Jaccard Index by 17%, 26%, and 15%, respectively, demonstrating the effectiveness of integrating prior information for community detection. Similarly, compared with CDGAAE_mod on Facebook 1684, CDGAAE shows improvements of 10%, 15%, and 10% in ONMI, F1, and the Jaccard Index, respectively, confirming that the introduction of the modularity optimization enhancement module leads to better community detection performance.

#### 4.4.4. Parameter Settings

We utilize Facebook 1684 as the experimental dataset, fixing the quantity of prior information at 2% in order to investigate the sensitivity of γ and λ in objective function of CDGAAE (Equation (19)). The weights of the semi-supervised clustering module and the modularity optimization enhancement module are controlled by the parameters γ and λ, respectively. We test these two parameters in the range {0.001, 0.01, 0.1, 1, 10, 100} in this part, and we use ONMI, F1, and the Jaccard Index to assess the results.

**Figure 5:**
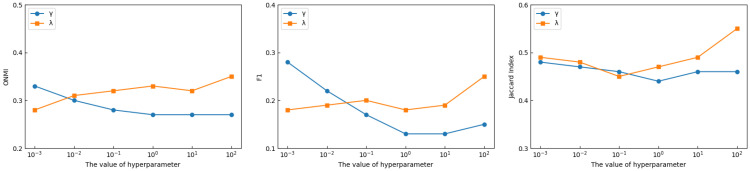
Comparison of performance of CDGAAE with different γ and λ.

The ONMI, F1, and Jaccard Index scores alter as γ and λ values change, as seen in Figure 5. The model performs best on the Facebook 1684 dataset when γ and λ are set to 0.001 and 100, respectively. It is crucial to strike a balance between the semi-supervised clustering loss, the modularity optimization enhancement loss, and the reconstruction loss of the graph structure and node characteristics. Thus, we set γ=0.001 and λ=100 in our experiments.

**Figure 6:**
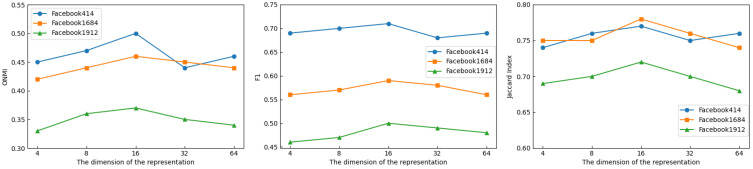
Comparison of performance of CDGAAE with different dimensions of the embedding.

We fix the fraction of prior knowledge at 2%, set the range of node representation dimension to {4, 8, 16, 32, 64}, and set the values of γ and λ to 0.001 and 100 in order to examine the effect of node representation dimension on model performance. Figure 6 displays the outcomes of the experiment.

The performance of CDGAAE exhibits comparable sensitivity to the dimensionality of node representation on each dataset, as illustrated in Figure 6. As the complexity of the node representation rises, the ONMI, F1, and Jaccard Index scores first rise and subsequently fall. On all three datasets, CDGAAE achieves the highest ONMI, F1, and Jaccard Index scores when the node representation dimensionality reaches 16. This demonstrates how important it is to select an appropriate node representation dimensionality. The network’s implicit information will be lost if the representation dimensionality is too low. If not, there will be a lot of noise in the node representation learning.

## 5. Conclusions

In this paper, a semi-supervised overlapping community detection method (CDGAAE) based on graph attention autoencoder is proposed to more accurately detect overlapping communities in vehicle social networks. The graph attention autoencoder effectively combines topological and attribute information while dynamically assigning different weights to each node. In addition, prior information is more efficiently integrated. Finally, a unified objective function is introduced to combine the graph structure and node attribute reconstruction loss, modularity optimization enhancement loss and semi-supervised clustering loss. Experimental results show that CDGAAE outperforms other methods on multiple datasets, demonstrating its effectiveness.

A semi-supervised overlapping community detection method based on graph attention autoencoder developed in this paper identifies overlapping community structures in VSNs by combining network topological information and node attribute information. The design of CDGAAE can play an important role in vehicle social network applications. First, communication efficiency is optimized. By identifying community structures, communication delays between vehicles can be reduced and communication resource allocation can be optimized. Second, community detection helps to optimize vehicle grouping, thereby improving the efficiency of ITS tasks such as path planning and fleet management. In addition, it can also improve privacy and security. By dividing communities, more accurate group encryption and privacy protection can be achieved. However, there are still many challenges in actual deployment, including limited computing resources, dynamic network topology, real-time requirements, etc. We will improve the practical applicability of our method in subsequent research.

Although this study focuses on static vehicle social networks and the proposed method is only applicable to stationary vehicle nodes, we recognize the importance of dynamic topology in real-world scenarios. In most cases, vehicle nodes are in dynamic motion. Therefore, our future work will address the community detection problem in dynamic vehicle social networks to adapt to the frequent changes in network structure and ensure scalability and adaptability in dynamic environments.

## Figures and Tables

**Table 1:** Notations and their descriptions.

Notations	Descriptions
*G*	The given vehicle social network
*V*	The set of nodes in vehicle social network
$v_{i}$	The *i*-th node in vehicle social network
*E*	The set of edges in vehicle social network
$e_{ij}$	The edge between $v_{i}$ and $v_{j}$ in vehicle social network
*X*	The node attribute matrix of vehicle social network
*A*	The adjacency matrix of vehicle social network
*n*	The number of nodes in vehicle social network
*m*	The number of edges in vehicle social network
$W^{\left(l\right)}$	The weight parameters of *l* attention heads
*k*	The number of communities
$\alpha_{ij}^{\left(l\right)}$	The normalized attention coefficient of *l* attention heads
$z_{i}$	The potential representation of node $v_{i}$
$d_{i}$	The degree of node $v_{i}$
$h_{i}^{\left(l\right)}$	The feature output of each attention head
*H*	Final community node representation matrix
$P_{ij}$	The state transition probability matrix between node $v_{i}$ and $v_{j}$
*Y*	The prior node community label matrix
$C_{i}$	The *i*-th community

**Table 2:** Statistics of datasets.

Dataset	Node	Side	Feature	Communities
Facebook414	159	27,082	105	7
Facebook1684	792	28,048	319	17
Facebook1912	755	60,050	480	46

## Data Availability

The data analyzed during the current study are available from the corresponding author upon reasonable request.
